# Climate and Processing Effects on Tea (*Camellia sinensis* L. Kuntze) Metabolome: Accurate Profiling and Fingerprinting by Comprehensive Two-Dimensional Gas Chromatography/Time-of-Flight Mass Spectrometry

**DOI:** 10.3390/molecules25102447

**Published:** 2020-05-24

**Authors:** Federico Stilo, Giulia Tredici, Carlo Bicchi, Albert Robbat, Joshua Morimoto, Chiara Cordero

**Affiliations:** 1 Dipartimento di Scienza a Tecnologia del Farmaco, Università degli Studi di Torino, 10125 Turin, Italy; federico.stilo@unito.it (F.S.); giulia.tredici@edu.unito.it (G.T.); carlo.bicchi@unito.it (C.B.); 2 Department of Chemistry, Tufts University, Medford, MA 02155, USA; joshua.morimoto@tufts.edu

**Keywords:** comprehensive two-dimensional gas chromatography, time-of-flight mass spectrometry, untargeted-targeted UT fingerprinting, tea metabolome, tea processing, climate events

## Abstract

This study applied an untargeted–targeted (UT) fingerprinting approach, based on comprehensive two-dimensional gas chromatography-time-of-flight mass spectrometry (GC×GC-TOF MS), to assess the effects of rainfall and temperature (both seasonal and elevational) on the tea metabolome. By this strategy, the same compound found in multiple samples need only to be identified once, since chromatograms and mass spectral features are aligned in the data analysis process. Primary and specialized metabolites of leaves from two Chinese provinces, Yunnan (pu′erh) and Fujian (oolong), and a farm in South Carolina (USA, black tea) were studied. UT fingerprinting provided insight into plant metabolism activation/inhibition, taste and trigeminal sensations, and antioxidant properties, not easily attained by other analytical approaches. For example, pu′erh and oolong contained higher relative amounts of amino acids, organic acids, and sugars. Conversely, black tea contained less of all targeted compounds except fructose and glucose, which were more similar to oolong tea. Findings revealed compounds statistically different between spring (pre-monsoon) and summer (monsoon) in pu′erh and oolong teas as well as compounds that exhibited the greatest variability due to seasonal and elevational differences. The UT fingerprinting approach offered unique insights into how differences in growing conditions and commercial processing affect the nutritional benefits and sensory characteristics of tea beverages.

## 1. Introduction

Comprehensive two-dimensional gas chromatography combined with time-of-flight mass spectrometry (GC×GC-TOF MS) is now considered one of the most informative chemical analysis techniques to characterize complex fractions in food [1,2,3,4]. The detailed profiling of known compounds (i.e., targeted analytes) can be extended from two- to five-fold compared to single dimension (1D) GC–MS analyses [2,5,6]; moreover, two-dimensional (2D) separation patterns can be investigated through new chromatographic fingerprinting algorithms, mass spectral features, and effective work-flows [7,8]. 2D chemical patterns can be treated as a unique sample′s fingerprint for the classification and cross-comparative analysis process [9]. Importantly, chromatographic fingerprinting has the intrinsic potential of being an accurate profiling strategy, since compound identity, made through MS signatures and relative retention (or indexes), provides excellent quantitation due to the accuracy of the detector response.

Key analytical features of GC×GC include: (a) higher separation power and enhanced resolution realized by combining orthogonal stationary phases for each separation dimension [10]; (b) improved sensitivity, by band focusing-in-space using cryogenic modulation; and (c) ordered/structured separation patterns for homologous series and chemically related organic compounds that aid in peak identification and structural elucidation. All of these characteristics make GC×GC-TOFMS the platform of choice to glean the highest level of information content encrypted in compounds/metabolites signatures, while, at the same time, provide reliable and robust results in the challenging domain of food chemical fingerprinting, traceability and origin authentication, technological impact, health, and aroma quality [2,5,11,12,13,14]. Since the fingerprint of multiple samples analyzed by GC×GC-TOF MS can be stacked prior to determining compound identity, compounds present in multiple samples only need to be identified once, which allows for automated peak area (or peak volume) quantitation of that compound in each sample.

In this study, we explore the complex tea metabolome and reveal compositional differences in farmer-processed green pu′erh teas from Yunnan Province (China), semi-oxidized oolong teas from Fujian Province (China), and fully fermented black teas from South Carolina (USA). Although the results reported herein compliment much wider studies [15,16,17] aimed at improving our understanding of how changes in climate conditions affect the sensory quality and nutritional benefits of tea, the primary objective of this study is to demonstrate that the untargeted–targeted (UT) fingerprinting and profiling approach we developed can be used to obtain information on both nonvolatile primary and secondary metabolites in differently processed teas collected over a two year period that are seasonally and elevationally different. Analytes include mono- and disaccharides, amino acids, and other low-molecular weight acids that constitute the primary metabolome. Phenolic acids, flavan-3-ols, methylxanthines, and hundreds of health beneficial and sensory active compounds arise from the specialized metabolome (previously called secondary metabolites).

In this paper, for the first time we combine untargeted and targeted fingerprinting analysis by template matching (i.e., UT fingerprinting) to comprehensively cover the detectable tea metabolome in the challenging context of abiotic stress induced by climate events. Then, we examine meaningful variations of known, targeted metabolites in detail to understand the synergic effect of external variables (i.e., processing, season, and elevation) on metabolite distribution and concentration. Findings are presented and discussed in the context of existing knowledge on tea plant reactions to external stimuli and/or in view of the potentials offered by a comprehensive data workflow process. Our primary aim, therefore, is to measure the relative distribution and concentration differences of primary and secondary metabolites in three different processed teas, namely, pu′erh, oolong, and black tea and to discuss the differences found in them.

## 2. Results and Discussion

We divided this section into two parts to provide a concise description of the experimental results, their interpretation, and conclusions drawn from them. First, we described how to access the 2D separation space to create an untargeted and targeted template by fingerprinting nonvolatile (derivatized) compounds detected by GC×GC-TOF MS. Then, a target analysis of primary and specialized metabolites was carried out to examine the effects of processing, rainfall, and temperature on specific biomarkers.

### 2.1. 2D Peak Patterns Complexity and Information Dimensions

**Figure 1:**
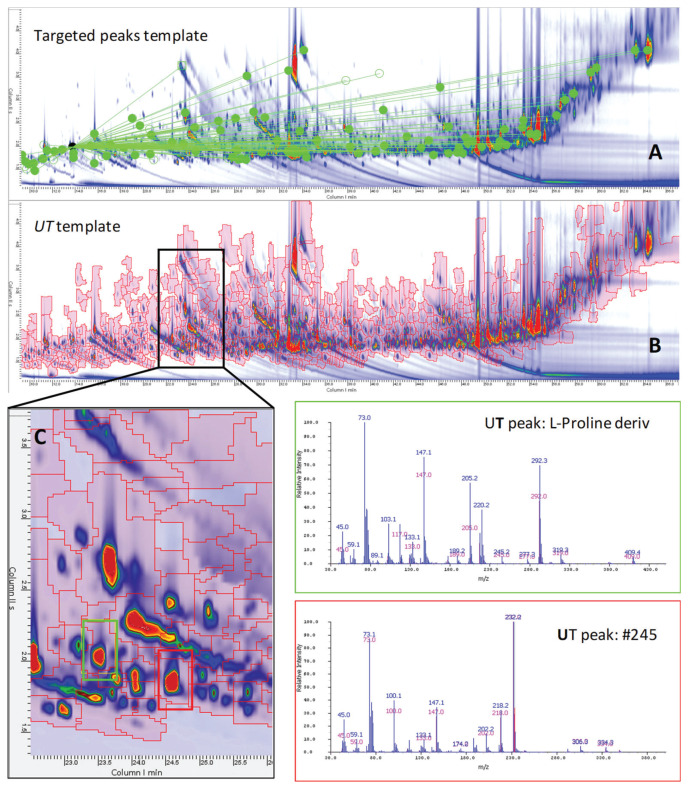
Contour plot of a Yunnan sample harvested in 2014 from high elevation and harvested after the monsoon season (2014_YUN_HM). (**A**) The distribution of targeted 2D peaks (green circles) while connection lines point to the IS (i.e., 1,4-dibromobenzene). (**B**) The distribution of untargeted–targeted (UT) peak-regions (red graphics) comprehensively covering the chromatographic space. Enlarged area of (**C**) highlights the position of the targeted peak (green squared) l-Proline and of the untargeted feature #245. Corresponding spectra are also reported.

As illustrated by the 2D peak patterns, the tea metabolome is rather complex. Figure 1A,B shows the contour plot of a Yunnan (YUN) tea sample harvested in 2014 from a high elevation (H) farm harvested during the monsoon (M, summer) season (2014_YUN_HM). The number of detected 2D peaks with a signal-to-noise ratio (SNR) above 100 [14] was approximately 900, covering 97% of the total response, while those above 400 SNR were 616, corresponding to 87% of the total response.

**Figure 2:**
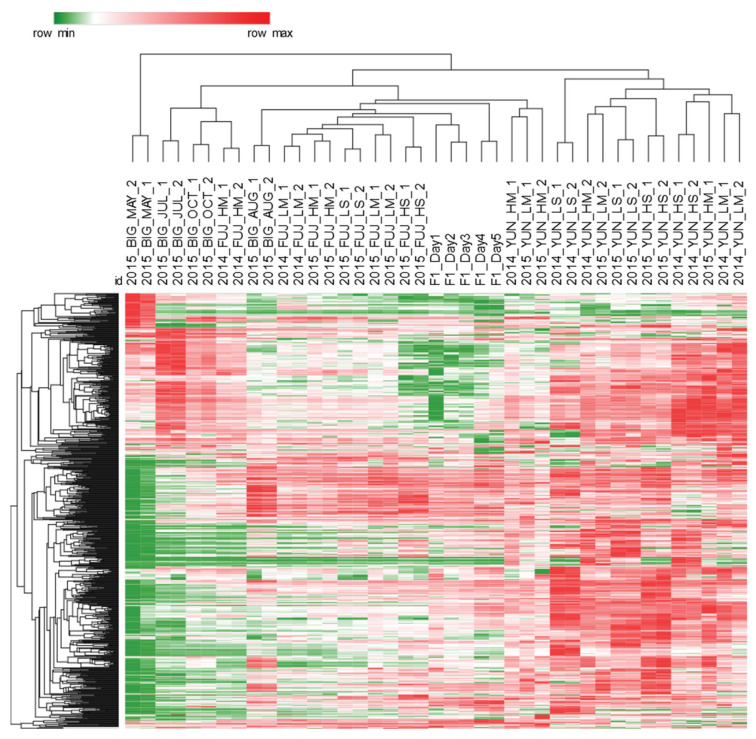
Heat-map visualization of % response data for 760 UT peak-regions cross-aligned on all samples′ patterns (41 samples including quality controls (QCs)). Hierarchical clustering was based on Euclidean distance metric and was performed after Z-score normalization of the data. Sample acronyms are reported in Section 4.2. Samples clustering, although incomplete, is guided by processing: Yunnan-pu′erh teas contains an overall higher abundance of primary metabolites, as highlighted by the predominance of red spots, and also Fujian-oolong teas, despite of some misclassified samples, are clustered independently from Bigelow-black tea.

A UT template of untargeted and targeted features was created to comprehensively map the detectable metabolome of tea samples. The template collects untargeted and targeted features together with their metadata (i.e., retention times, MS spectral signatures, etc.) while enabling their cross-alignment within samples patterns. The procedure, illustrated in the experimental section (Section 4.8.) and visualized in Figure S1, enables effective and reliable cross-alignment of 2D peaks and peak-regions across all sample patterns. The resulting data matrix was 41 × 760 dimensional (i.e., samples × UT peak-regions) and its information potential is visualized in the heat-map in Figure 2. The graphical rendering represents the UT peak-region % response distribution; hierarchical clustering (HC) is based on Euclidean distances and was obtained after Z-score (mean subtraction and division by standard deviation) normalization of the data.

Processed tea samples were clustered according to their detectable peak regions. For example, pu′erh tea contains a higher abundance of primary metabolites, as evidenced by the predominance of red spots, with post-fermentation most likely increasing the amount of free amino acids, organic acids, and sugars. Targeted profiling confirmed this hypothesis (see Section 2.2.). Conversely, black tea and the semi-oxidized oolong tea were clustered nearly independently despite misclassification of some samples, see biological replicates of 2014_FUJ_HM and 2015_BIG_AUG. Finally, quality control (QC) samples (F1_Day#) show similar trends and were closely clustered in the middle of the graph.

Although differences in primary and specialized metabolite distributions, based on commercial processing methods was expected, our interest was mostly directed to the impact that climate had on specific, diagnostic analytes. Therefore, the response from targeted features was extracted from the UT data matrix and analyzed in more detail.

### 2.2. Targeted Features Distribution according to Processing

First, the targeted compound distribution was observed as a function of each samples characteristic processing. The list of targeted analytes together with their retention times in two dimensions (*^1^t_R_*, *^2^t_R_*), and *I^T^_s_* (experimental and reference/tabulated) is reported in Table 1. Analytes were identified based on criteria detailed in Section 4.8.

Coverage of the primary metabolome separation space was very good and informative, since it was possible to map the distribution of 15 amino acids, 13 sugars including mono- and di-saccharides and sugar acids, as well as the 21 organic acids mainly involved in cell metabolism. In addition, the secondary metabolites included methylxanthines (caffeine and theobromine), flavan-3-ols (catechin, epicatechin, gallocatechin, and epigallocatechin), and phenolic acids (quinic, gallic, caffeic, and chlorogenic acid).

The high information content encrypted in the tea data provides a deeper level of understanding as to how abiotic stress factors, e.g., rainfall and temperature, affect plant metabolism activation/inhibition, producing compounds that either directly or indirectly (due to processing) affect the sensory (mainly taste and trigeminal sensations) and nutritional quality (amino acids, antioxidants, etc.) [16] of brewed tea and consumer willingness to buy. For example, processing is the most influential variable affecting the distribution and relative amount of metabolites; as evidenced by UT features distribution (Figure 2), where unfermented teas (Yunnan–pu′erh) were discriminated for their higher relative abundance of detected metabolites.

**Figure 3:**
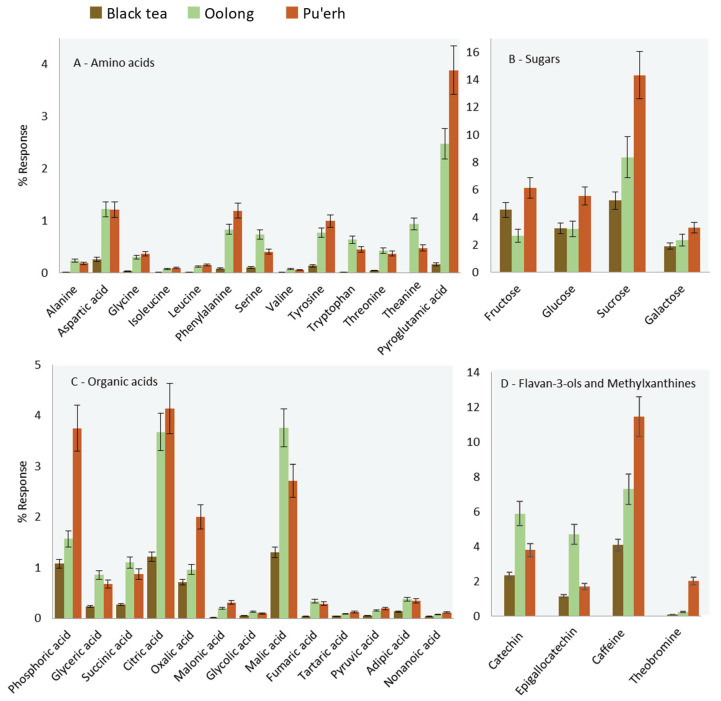
Histograms illustrating the relative distribution (% response) for targeted features based on chemical classes (**A**) amino acids, (**B**) sugars, (**C**) organic acids, (**D**) catechins, and methylxanthines). Error bars correspond to ± SD over all samples belonging to the same class.

Figure 3 histograms illustrate the relative distribution (% response) of selected chemical classes (amino acids, sugars, organic acids, flavan-3-ols, and methylxanthine) across all samples.

**Figure 4:**
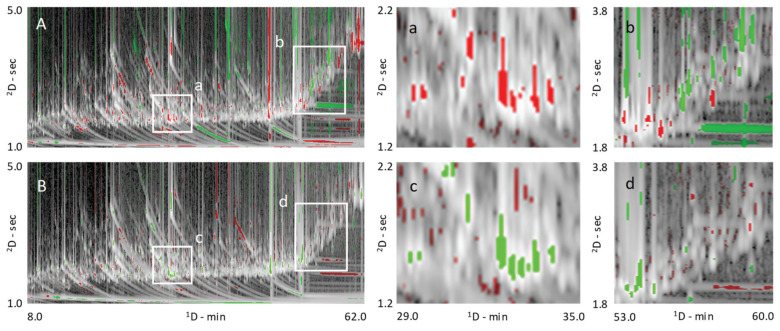
Pair-wise comparison based on visual features fingerprinting [7], the comparative visualization is rendered with a colorized fuzzy difference. Pixels brightness indicates the magnitude of the absolute response; pixels hue indicates whether the analyzed image (i.e., 2015_YUN_HS in (**A**) or 2015_FUJ_LS in (**B**)–green) or reference image (i.e., 2015_FUJ_HS for both visualizations-red) has the higher response value. Pixel saturation (color vs. grey tones) indicates the magnitude of the difference between the analyzed and reference images, with grey indicating equal pixel values and bold colors indicating large differences. Enlarged areas, corresponding to white rectangles, highlight absolute compositional differences for monosaccharides (a/c) and of some secondary metabolites belonging to the chlorogenic acid and flavan-3-ol classes (b/d).

Except for fructose and glucose derivatives, black tea obtained from a farm in South Carolina, USA, contained lower amounts of targeted analytes and, in this regard, were similar to oolong teas from Fujian, China. The fermentation/oxidation process that occurs during tea production explains this finding and can be seen also by the pair-wise comparison based on visual features between Fujian and Yunnan teas. The comparative visualization rendered with a colorized fuzzy difference is shown in Figure 4A. In the visualization, the brightness of the pixels indicates the magnitude of the absolute response; pixel hues indicate whether the analyzed image (i.e., 2015_YUN_HS, green) or reference image (i.e., 2015_FUJ_HS red) has the higher response value. The saturation (color vs. grey tones) of a pixel indicates the magnitude of the difference between the analyzed and reference images, with grey indicating equal pixel values and bold colors large differences. Enlarged areas, corresponding to white rectangles, in Figure 4A highlight absolute compositional differences for monosaccharides (a) and some of the secondary metabolites corresponding to the chlorogenic acids and flavan-3-ols chemical classes (b).

Oolong tea, according to processing practices, undergoes partial fermentation, while pu′erh tea does not [18]. According to the literature, minimally fermented teas are richer in amino acids, which is consistent with the data shown in Figure 3A. Pyroglutamic acid is of interest within the target class of amino acids because it is the biosynthetic precursor of theanine [19,20]. Theanine is described as the compound responsible for the brothy-sweet-umami taste typical in green teas [18,21]. Leucine, isoleucine, valine, and phenylalanine are also of interest because of their role as precursors of key-aroma compounds in tea [22,23]. Their lower abundance in black tea samples can be explained by the Strecker degradation, which converts α-amino acid into aldehydes such as 2- and 3-methylbutanal, 2-methylpropanal, and phenyl acetaldehyde. These potent odorants, with their malty, buttery, floral, and honey-like notes are generally more abundant in black teas [24,25,26].

Oolong and pu′erh teas are, on average, richer in mono and di-saccharides compared to black teas, as illustrated by the histograms in Figure 3B. As highlighted in the comparative visualization of Figure 4A (a-zoomed area), between them, the pu′erh harvested in 2015 at high elevation during spring (2015_YUN_HS) showed higher amounts of fructose and glucose derivatives compared to the oolong (2015_FUJ_HS). According to the literature [27], a possible explanation is the fermentation process. On the one hand, fermentation induces the release of sugars and other metabolites and promotes their degradation via cell metabolism. The manufacturing process for Pu′erh tea produces a sugar profile more similar to that of semi-fermented (oolong) teas [27]. This result could be due to fermentation that is partially blocked post-harvest and/or because sugars are energy substrates for the microorganisms involved [27].

Considering organic acids, in Figure 3C, citric, malic, oxalic, phosphoric, and succinic acids were the most abundant in all of the teas [28]. Here as well, the fermentation process induced in black teas affects these metabolites by substantially reducing their concentration. In contrast, the organic acids profile in oolong and pu′erh teas were similar except for citric, oxalic, and phosphoric acids, which were more abundant in pu′erh compared to oolong tea, while malic and succinic acids were more abundant in oolong teas.

Finally, flavan-3-ols derivatives and methylxanthines showed interesting trends as illustrated in Figure 3D. These analytes belong to the group of secondary metabolites with known biological activity [29] and inform about tea quality [30,31,32] and processing practices [33]. The fermentation process, for example, significantly reduces the levels of catechins by promoting the formation of dimers and oligomers such as pro-anthocyanidins, theaflavins, and thearubigins [34]. Oolong teas, as expected, revealed a higher relative amount of flavan-3-ols, while Pu′erh tea shows higher levels of methylxanthines [18,21,29,33].

### 2.3. Targeted Features Distribution according to Climate Events and Elevation

First, this section examines in detail the effects of water availability as a function of seasonal rainfall, namely, spring (pre-) and summer (post-monsoon), variations. Second, we examine the effects of elevation to learn how differences in temperature affect metabolite distributions in the samples analyzed. Since the samples were collected from the same farms at the same time but different elevations, differences in temperature provide insight into how metabolite distribution and concentration can change depending on the time of harvest on any given day. Climate strongly affects crop quality and plant development by altering the distribution of primary and specialized metabolite signatures [16,35]. For this purpose, pu′erh and Oolong tea samples are intercompared for two harvest years (2014 and 2015); samples were harvested from the same farm in Yunnan and Fujian during the spring season (S) and after the onset of the monsoon rains (M) in plots located at different elevations.

To exclude the effect of processing, the pu′erh and oolong metabolite signatures were examined separately, to look for potentially informative biomarkers that could help to explain the plant-climate effects of drought (S) and heavy rain (M). Informative metabolites from the two groups were matched and those in common retained as robust biomarkers. Sample groups from the two harvest years were investigated by a non-parametric test, viz., Kruskal–Wallis, chosen for selecting metabolites that show statistically meaningful differences in distribution (% response was used as a quantitative indicator) at a confidence interval of 95%, between monsoon and spring seasons in each crop. Subsequently, a partial least square discriminant analysis (PLS-DA) was applied. This function combines dimensionality reduction and discriminant analysis, not assuming the data to fit a particular distribution [36]. Variables having variable importance in the projection (VIP) values ± SD greater than 1 were considered statistically meaningful to describe the impact of water availability (or elevation in the second case) on the tea metabolome. The match between variables from the Kruskal–Wallis test and PLS-DA provides a list of potential markers with a relevant informative role in describing the phenomenon under study.

**Figure 5:**
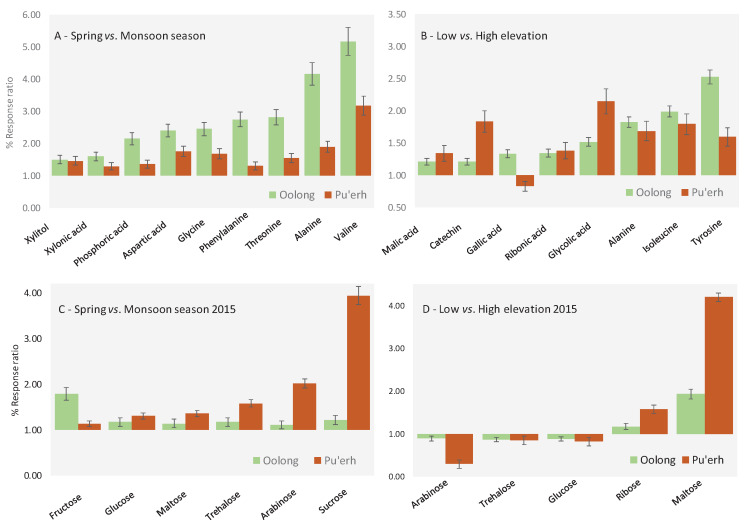
Histograms illustrating the % response ratio between the spring vs. monsoon season (**A**) and low vs. high elevation (**B**) teas. Error bars correspond to ± SD over all samples belonging to the same class. The sugar class is examined independently and results visualized in Figure (**C**) and (**D**) for seasonal and elevation effects respectively.

Seasonal rainfall variations are one of the most important sources of abiotic stress in tea plants [16]; the most informative metabolites in common for both teas were: alanine 3TMS, aspartic acid 2TMS, glycine 3TMS, threonine 3TMS, valine 2TMS, phenylalanine 3TMS, phosphoric acid 3TMS, xylonic acid 2TMS, and xylitol 3TMS. The Figure 5A histogram shows the ratio of the average percent response between the spring and monsoon seasons. Values above 1 indicate an up-regulation of the specific metabolite in the spring season. Colors indicate tea groups (oolong is green and pu′er light brown).

Based on our previous work, we were not surprised to find the targeted metabolites more abundant in spring samples. This evidence could be due to mass differences between the spring and summer harvest [17,37]. Note: rainfall 10-days prior to the spring harvest was zero in Yunnan and 30–50 mm in Fujian, while rainfall 10-days prior to the summer harvest was between 300 and 500 mm in both provinces. Unlike the volatile metabolites, where two-thirds of the 500 metabolites detected increased/decreased by more than 60%, even within the same chemical family, primary metabolite concentrations trended in the same direction. The results reported herein are consistent in that spring tea harvested at high elevation contained more and higher concentrations of the sensory pleasing, health beneficial volatile, secondary metabolites than did all other samples [15] and is in agreement with farmers′ perceptions of quality [16].

In particular, six of the nine compounds showing meaningful variations between the spring and monsoon season (Figure 3A) are amino acids. The literature provides various hypotheses and interpretations for the higher abundance of amino acids in spring teas. Lee et al. [38] found direct correlation in green tea between the amino acid content and young leaves harvested in spring, while Qu et al. [39], Zeng et al. [40], and Upadhyaya et al. [41] believe stress due to moderate drought caused the increase in amino acid concentrations. We confirmed the findings of these studies for both oolong and pu′erh teas and suggest that the higher alanine concentration in spring tea is due to the stimulation of theanine formation, because of the conversion of alanine in ethylamine, which is a precursor in theanine biosynthesis [38]. The role of water deficit tolerance is also exerted on phenylalanine [42,43], while valine is an aroma precursor of 2-methylpropanal, responsible for fresh, herbal, and green aroma notes. The literature also reports higher levels of aspartic acid and threonine in spring teas [38,39]. We report for the first time upregulation of glycine in teas harvested in the spring season.

Within the organic acids class, a meaningful variation was observed for xylonic acid, which is produced by *Gluconobacter oxydans* fermentation of xylose [44,45]. Xylonic acid was up regulated in both oolong and pu′erh teas, as was phosphoric acid, known to be modulated by both drought and threonine stress [46,47]. Moreover, its presence could be influenced by *Fusarium* development, improving water uptake and nutrition by elevating phosphate supply [47]. We also found that xylitol was upregulated but, to the best of our knowledge, the literature lacks information on its role in the abiotic stress of plants.

Regarding sugars, the literature reports a general increase of sucrose, glucose, fructose, and maltose in many plants stressed due to drought [48], but with the sample set under study, harvest year impact confounded this phenomenon. In particular, by comparing 2014 spring vs. 2015 spring and 2014 monsoon vs. 2015 monsoon, 2014 seasons were richer in overall sugar content, respectively, with 17% ± 3% for spring and 15% ± 2% for the monsoon season. Targeted sugars were examined by comparing the response data for samples harvested in the same year (i.e., 2015). Results are summarized as a response ratio in Figure 5C for seasonal and in Figure 5D for elevation effects. Seasonal findings reveal six significant compounds, namely, fructose, glucose, maltose, trehalose, arabinose, and sucrose. These sugars are more abundant in spring samples but, interestingly, with the exception of fructose, differences in content are substantially more marked in pu′erh samples.

With regard to elevational effects, an approximately linear function exists between elevation and temperature: air temperature decreases linearly with an increase in elevation at a rate of between 4.0 and 8.1 °C/Km, depending on territorial conformation, which is more pronounced during summer compared to winter months [49,50]. As above, we considered Oolong—Fujian and pu′erh—Yunnan teas separately with supervised statistics revealing robust common biomarkers. Although “high” and “low” elevations are different for the two provinces, in Yunnan, high elevation corresponded to 1790 m and low, 1180 m, while in Fujian, high elevation teas were 690 m and low, 112 m. Despite this difference, together with latitude, on absolute temperatures and daily variations, the differential elevation is comparable for the two locations, i.e., 610 m and 578 m for the Yunnan and Fujian farms, respectively.

The most meaningful metabolites resulting from independent elaboration of Yunnan and Fujian samples applying the Kruskal–Wallis and PLS-DA statistics include eight metabolites: alanine 3TMS, isoleucine 2TMS, tyrosine 2TMS, catechin 5TMS, gallic acid 2TMS, glycolic acid 2 TMS, malic acid 3TMS, and ribonic acid TMS. Figure 5B shows the histogram of the ratio of average % response between low and high elevation samples. Values above 1 indicate an up-regulation of specific metabolites from high elevation teas, where oolong is in green and pu′erh light brown.

For the most relevant metabolites, the trend is coherent and indicates an up-regulation of the targeted analytes in low elevation samples. Similar to that found for rainfall, amino acids play a role in discriminating elevational effects. Our findings are consistent with that reported in the literature, indicating temperature (in addition to rainfall and sun exposure time) as a key-factor affecting amino acids accumulation [38]. Here, three of them, namely alanine, isoleucine, and tyrosine, are more abundant in high compared to low elevation teas. Very interestingly, alanine was also informative of drought stress resulting in up-regulation in both oolong and pu′erh teas from the spring harvest.

Catechin is a specialized metabolite belonging to the group of flavan-3-ols, and is more abundant in low elevation teas [35]. This metabolite, and in general flavanols, is known to contribute to the bitter taste and astringency [38,51] of brewed tea and, together with theanine, plays a major role in the nutritional quality of tea. This characteristic metabolite signature is in accordance with farmers′ perceptions that high elevation teas have a higher aromatic quality with sweet, fruity, and floral notes, while low elevation teas are generally characterized by a greener, herbal, bitter taste [35].

Gallic acid, a specialized metabolite belonging to the phenolic acid class [34], and glycolic, malic, and ribonic acids were all up-regulated in low elevation teas, with gallic acid being the only exception in pu′erh samples. Besides its specific physiological role in the adaptation to temperature profiles, gallic acid has an additional health benefit due to its antioxidant properties and bioavailability [29,30,31,52].

No solid and convincing data is found in the literature for sugars regulation, which is consistent with our data, see also Figure 5D. Nevertheless, arabinose, trehalose, glucose, ribose, and maltose show a significant variation between low and high elevation in the 2015 samples. In particular, arabinose, trehalose, and glucose are down-regulated in low elevation samples, while ribose and maltose are up-regulated. As observed for seasonal effects, sugars variations are more marked in pu′erh samples. The comparative analysis shown in Figure 4B illustrates the colorized difference in metabolite distribution between an oolong tea from 2014 harvested at low elevation in spring (analyzed image) compared to samples harvested at high elevation (reference image). Sugars, in particular the monosaccharides (Figure 4c-zoomed area), resulted in up-regulation in the low elevation sample (green colored pixels). In contrast, the differential distribution of some specialized metabolites belonging to the chlorogenic acid class did not show meaningful differences between these two-pairs (Figure 4d, zoomed area).

## 3. Conclusions

In this study we explored the information content of the GC×GC-TOF MS data in delineating metabolite signatures capable of capturing the impact of processing and growing conditions (rainfall and temperature) in tea (*Camellia sinensis* L. Kuntze) over a two-year period. Unlike our previous work on unprocessed tea, which focused solely on volatile secondary metabolites and metals, this work′s objective was to identify key primary metabolites and other biomarkers that can contribute to our understanding of the complex relationships and feedback loops that occur between human and natural systems, specifically, strategies to counter climate effects on tea plants. To obtain these biomarkers we developed and validated a work-flow template capable of accurately mapping metabolome variations while providing a very large set of metadata that covered both targeted and untargeted features.

Based on the findings described herein, we found that alanine, aspartic acid, glycine, threonine, valine, phenylalanine, phosphoric acid, xylonic acid, and xylitol were all up-regulated by the plant in the spring compared to summer, monsoon impacted teas. Although harvest year confounded sugar results, some monosaccharides including fructose, glucose, maltose, and arabinose were also higher in concentration in spring teas. Differences in elevation also affected metabolite distribution. For example, low elevation leaves contained more alanine, isoleucine, tyrosine, catechin, gallic acid, glycolic acid, malic acid, and ribonic acid than high elevation tea. These results were independent from processing; suggesting rainfall and temperature affect specific metabolic pathways, with future work aimed at revealing these effects.

Untargeted features, due to the rich set of metadata (retention times in two dimensions, MS fragmentation patterns, absolute and relative responses, etc.) can be ex post explored, for their information potential in the discrimination of samples with compound identity elucidated by combining MS spectral signature at 70 eV, *I^T^* and pattern relative position. Ex post identification is enabled with high confidence because of the high separation efficiency provided by the combination of band compression in space from thermal modulation and differential selectivity (i.e., orthogonality [10]) between separation dimensions.

## 4. Materials and Methods

### 4.1. Chemicals and Reference Solutions

Pure standards of *n*-alkanes (from *n*-C9 to *n*-C30) for system evaluation and linear retention indexes (*I^T^*), internal standards (ISs) 4-chlorophenylalanine and 1,4-dibromobenzene were from Merck (Milan, Italy).

The mixture of *n*-alkanes for the *I^T^* solution was prepared in cyclohexane at a concentration of 100 mg/L; internal standards (ISs) solutions were prepared in dichloromethane (GC grade) at a concentration of 4 mg/mL.

Pure standards for identity confirmation of pyruvic acid, malonic acid, phosphoric acid, succinic acid, glyceric acid, fumaric acid, malic acid, citric acid, Alanine-Ala, Aspartic acid-Asp, Glutamic acid-Glu, Glycine-Gln, Isoleucine-Ile, Leucine-Leu, Methionine-Met, Phenylalanine-Phe, Proline-Pro, Serine-Ser, Threonine-Thr, Tryptophan-Trp, Tyrosine-Tyr, Valine-Val, glycerol, xylitol, mannitol, myo-inositol, fructose, glucose, and the internal standards (ISs), 4-chlorophenyl alanine (quality control–QC for derivatization), and 1,4-dibromobenzene (QC for GC normalization) were purchased from Merck (Milan, Italy).

Derivatization reagents and HPLC grade solvents: O-methyl hydroxylamine hydrochloride (MOX), (*N*,*O*-bis(trimethylsilyl)trifluoroacetamide (BSTFA) methanol, pyridine, *n*-hexane, cyclohexane, dichloromethane, and toluene were also obtained from Merck.

### 4.2. Tea Samples

Tea samples were a subset of those analyzed in the Kfoury et al. [17] study and belong to the same tea specimen that was ultimately transplanted in Yunnan and Fujian (China) and South Carolina (SC, USA). QC samples were from a commercial batch of black tea available in the author′s laboratory. Tea samples at each location were collected from the same farm, but at different elevation and harvested pre- (spring, S) and post-monsoon (summer, M) seasons in 2014 and 2015 as detailed in Table 2. Spring tea in Yunnan corresponds to drought conditions (0 mm rainfall), while tea plants in Fujian experienced some rainfall (50 mm), whereas monsoon rains produced between and 300 and 500 mm of rainfall within a 10-day period prior to harvest.

After harvest, tea leaves were subjected to different processing to obtain pu′erh tea from Yunnan Province, oolong tea from Fujian Province, and black tea from South Carolina.

Tea leaves were harvested at different elevation in 2014 and 2015.

- Yunnan (YUN) is a post-fermented pu′erh tea collected in the spring (S) and summer (monsoon, M) at 1180 m height (low elevation, L) and 1790 m (high elevation, H).
- Fujian tea (FUJ) is a semi-oxidized oolong tea collected in the spring and monsoon seasons at 112 m (L) and 690 m (H).
- Bigelow (BIG) is an oxidized black tea from a farm in South Carolina (USA). Teas were harvested in 2015 in May, July, August, and October. Rainfall and temperature were 21 ± 3 °C and 105 mm, 28 ± 2 °C and 97 mm, 28 ± 2 °C and 188 mm, and 23 ± 3 °C and 453 mm, respectively. Note that the amount of rainfall is similar to that experienced by plants in China during the monsoon season. The elevation of the farm, located on Wadmalaw Island, South Carolina, is 7 m.

### 4.3. Primary Metabolites Extraction and Derivatization

#### 4.3.1. Extraction

An aliquot of 0.500 g of dried tea leaves, carefully milled to obtain a fine and homogeneous powder, was placed in a centrifuge glass tube with 5.0 mL of ultrapure water. Extraction was conducted at 70 °C and with the aid of ultrasound (US-40 KHz ± 5%) for 15 min. During method development and validation, each sample was extracted 5–10-times up to exhaustive extraction of a selection of targeted compounds. Results on extraction yields, estimated on absolute analytes responses between extraction #1 and #10 are reported in the supplementary material. Figure S2 illustrates a schematic diagram of the extraction/derivatization procedure while Figure S3 shows experimental results on targeted primary metabolites extraction yields. Based on experimental results, the first five extraction aliquots were collected together and submitted to oximation/silylation.

After hot water extraction, centrifugation was carried out at 5500 rpm for 10 min, the supernatant was then carefully collected and filtered with Nylon HPLC filters with 20 µm pores.

#### 4.3.2. Derivatization

Of the water extract 1.00 mL from the first five extractions were collected together, spiked with 20 µL of 4-chlorophenylalanine solution (4 mg/mL in dichloromethane), and dried under a gentle stream of nitrogen into 1.5 mL glass vials. Then, 45 µL of MOX solution (20 mg/mL in pyridine) were added followed by the metoximation reaction at 60 °C for 2 h. Lastly, 60 µL of (*N*,*O*-bis(trimethylsilyl)trifluoroacetamide—BSTFA were added to the reaction mixture. The silylation reaction was carried out at 60 °C for 1 h. At the end of the derivatization step, the reaction mixture was spiked with 5 µL of 1,4-dibromobenzene (IS 4 mg/mL in dichloromethane); an additional 90 µL of dichloromethane was added up to a final volume of 200 µL.

For primary metabolites identity confirmation, 1.00 mL of primary metabolites standards mixture (listed in Section 4.1.) was submitted to the derivatization procedure and analyzed under conditions described in Section 4.4.

### 4.4. GC×GC-TOF MS: Instrument Set-Up and Experimental Conditions

GC×GC analyses were performed on an Agilent 7890B GC (Agilent Technologies, Wilmington, DE, USA) unit coupled with a Bench TOF-Select™ system (Markes International, Llantrisant, UK). Electron ionization was set at 70 eV. The ion source and transfer line were set at 290 °C. The MS optimization option was set to operate in single ionization with a mass range between 35 and 550 *m*/*z*; data acquisition frequency was 100 Hz; filament voltage was set at 1.60 V.

The system was equipped with a two-stage KT 2004 loop thermal modulator (Zoex Corporation, Houston, TX, USA) cooled with liquid nitrogen controlled by Optimode™ V.2 (SRA Instruments, Cernusco sul Naviglio, Milan, Italy). The hot jet pulse time was set at 350 ms, modulation period was 5 s, and cold-jet total flow was progressively reduced with a linear function from 30% of the mass flow controller (MFC) at initial conditions to 5% at the end of the run.

### 4.5. GC×GC Columns and Settings

The column set was configured as follows: ^1^D DB-5 column (95% polydimethylsiloxane, 5% phenyl; 30 m × 0.25 mm *d*_c_, 0.25 μm *d*_f_) coupled with a ^2^D OV1701 column (86% polydimethylsiloxane, 7% phenyl, 7% cyanopropyl; 2 m × 0.1 mm *d*_c_, 0.10 μm *d*_f_), from J&W (Agilent, Little Falls, DE, USA). The first 0.80 m of the 2D column, connected in series to the ^1^D column by a silTite μ-union (Trajan Scientific and Medical, Ringwood, Victoria, Australia), were wrapped in the modulator slit and used as loop-capillary for cryogenic modulation. The carrier gas was helium at a constant flow of 1.6 mL/min. The oven temperature program was from 75 (1 min) to 290 °C (15 min) at 4 °C/min.

For primary metabolites profiling, 2.0 μL of the derivatized solution (Section 4.1) was analyzed under the following conditions: split/splitless injector in the split mode, split ratio 1:20, injector temperature 290 °C. The *n*-alkanes liquid sample solution for *I^T^* determination was analyzed under the following conditions: split/splitless injector in split mode, split ratio 1:50, injector temperature 290 °C, and injection volume 1 µL.

### 4.6. Method Performance Parameters: Retention Times and Response Repeatability

Method validation was run on a three-days over two-weeks basis and aimed at the evaluation of repeatability of retention times and UT peaks response intermediate precision [53].

Validation and verification procedures were in line with consolidated protocols for omics studies [3]. Retention times in both chromatographic dimensions (*^1^t_R_* and *^2^t_R_*) were collected from UT peak-regions above a % response of 0.020 for a total of 41 analytical runs and across all working days. Results were visualized in the scatter diagrams of Figure S4 as % relative standard deviation (% RSD). Quite good retention time stability was achieved with an average % RSD of 0.38 for *^1^t_R_* and 3.15 for *^2^t_R_*. Response repeatability was calculated on selected UT peak-regions (i.e., those with an average % resp. > 0.020) from QC samples acquired over five days in two-weeks. Results are visualized in the scatter diagram Figure S4 as % RSD. Repeatability was on average, over 760 UT peak-regions, 17.6% with a median of 15.9%. By observing performance for most representative UT peaks with a % resp. > 0.1% the statistics is even better and the average repeatability reaches the 13.5% RSD.

As indicator of analytes relative abundance, it was adopted the % response for UT peaks and peak regions; it was calculated based on normalized 2D volumes (vs. ISs) and referred to the total response calculated for UT peaks or peak-regions excluding interfering compounds and column bleeding [3].

### 4.7. Data Acquisition and 2D Data Processing Software

GC×GC data were acquired by TOF-DS software (Markes International, Llantrisant, UK) and processed using GC Image GC×GC Edition, ver. 2.9 (GC Image, LLC, Lincoln, NE, USA). Data elaboration and results visualization were by XL-Stat 2014 (Addinsoft Inc., New York, USA) and by open source Gene-E (Broadinstitute.org).

### 4.8. Combined Untargeted and Targeted (UT) Fingerprinting: Principles and Operative Steps

The 2D data elaboration workflow adopted to comprehensively map tea primary metabolites signatures is illustrated in Figure S1. It was developed for complex fractions of extra virgin olive oil volatiles [13] and then validated for its effectiveness in other applications including cocoa processing markers [54], urine metabolomics in diet-intervention studies [55], wine and tea volatiles [4,25,56], and metabolite fingerprinting of high quality hazelnuts [57]. It combines untargeted and targeted pattern recognition based on the template matching algorithm developed by Reichenbach et al. [58].

The work-flow includes the following steps:

1. Chromatograms preprocessing for background subtraction and 2D-peaks detection.
2. Untargeted feature template generation by cross-matching samples 2D-peaks templates. Re-alignment of 2D-peaks patterns and generation of a 2D peak-region features template.
3. Refining of the untargeted feature template by eliminating solvent, bleeding, and interfering peaks. Identification of target compounds by spectral similarity direct and reverse match factors (NIST similarity algorithm [59]–threshold values DMF 900–RMF 930) with commercial databases and ^1^D *I^T^* coherence (*I^T^* ± 10 units). Creation of a UT template with both untargeted and targeted features.
4. Application of the UT feature template to each sample and export metadata in the excel file for further data elaboration. The output is a data matrix of aligned 2D peaks and/or peak-regions and related metadata (^1^D and ^2^D retention times, compound names for target analytes, fragmentation pattern, single ions, or total ions response) available for comparative purposes and further processing [60,61,62].

Visual features fingerprinting, with pair-wise image comparison, also was performed with the “colorized fuzzy difference” rendering mode [63]. The algorithm computes the difference at each data datapoint (i.e., the output of the detector at a point in time) between pairs of TIC chromatograms. These differences are mapped into hue–intensity–saturation (HIS) color space to create an image for visualizing the relative differences between image pairs in the retention-times plane [63].

## Figures and Tables

**Table 1:** List of 74 targeted analytes together with retention times in first and second dimensions (*^1^t_R_*, *^2^t_R_*), and *I^T^* (experimental and reference NIST Chemistry WebBook, SRD 69 [https://webbook.nist.gov/chemistry/gc-ri/] values).

Chemical Class	Compound Name	*^1^t_R_* (min)	*^2^t_R_* (sec)	Exp. *I^T^*	Ref. *I^T^*
Amino acids	Alanine TMS	10.08	1.45	1103	1110
	Valine 2TMS	13.56	1.70	1212	1215
	Serine 2TMS	15.06	1.98	1260	1266
	Leucine 2TMS	15.95	1.85	1288	1294
	Threonine 2TMS	16.22	1.95	1298	1305
	Isoleucine 2TMS	16.23	1.77	1299	1306
	Glycine 3TMS	16.48	1.74	1305	1310
	Proline 2TMS	16.50	1.80	1305	1304
	Methionine TMS	20.25	2.36	1422	1417
	Aspartic acid 2TMS	20.50	2.21	1430	1427
	Pyroglutamic acid TMS	23.25	3.44	1519	1515
	Phenylalanine 2TMS	26.40	2.06	1626	1624
	Theanine TMS	29.05	3.44	1721	/
	Tyrosine 2TMS	33.53	3.33	1892	1900
	Tryptophan TMS	40.39	3.45	2180	2186
Organic acids	Hexanoic acid TMS	9.30	1.53	1075	1074
	Glycolic acid 2TMS	9.30	1.63	1075	1077
	Pyruvic acid 2TMS	9.63	1.60	1085	1085
	Oxalic acid 2TMS	10.99	1.97	1129	1133
	Hydroxybutyric acid TMS	11.77	1.66	1156	1158
	Malonic acid 2TMS	13.28	1.96	1204	1201
	Phosphoric acid 3TMS	15.41	2.22	1271	1267
	Succinic acid 2TMS	16.87	1.99	1317	1314
	Glyceric acid 3TMS	17.24	1.79	1328	1330
	Fumaric acid 2TMS	18.04	1.91	1353	1353
	Nonanoic acid TMS	18.36	1.74	1363	1368
	Ribonic acid TMS	19.44	2.40	1396	1398
	Malic acid 3TMS	22.29	2.01	1488	1490
	Adipic acid 2TMS	22.95	2.05	1509	1510
	Tartaric acid 4TMS	26.60	1.93	1633	1640
	Arabinonic acid TMS	26.62	2.27	1634	/
	Citric acid 4TMS	31.60	2.05	1817	1815
	Galactonic acid 6TMS	35.78	1.83	1981	1989
	Galactaric acid 6TMS	36.97	1.92	2031	2024
	Linoleic acid TMS	41.12	1.93	2214	2212
	Glucuronic acid 5TMS	44.31	1.93	2367	/
Polyalcohols	Glycerol 3TMS	15.38	1.59	1270	1278
	Xylitol 5TMS	28.15	1.65	1688	1692
	Arabinitol 5TMS	28.44	1.69	1698	1702
	Ribitol 5TMS	28.88	1.67	1714	1717
	Glucitol 6TMS	34.21	1.72	1919	1927
	Mannitol 6TMS	34.27	1.73	1921	1925
	Myo-Inositol 6TMS	38.06	1.91	2078	2073
Sugars	Threonic acid 4TMS	24.35	1.80	1556	1553
	Arabinose 4TMS	27.27	1.71	1657	/
	Ribose 4TMS	27.68	1.71	1671	1668
	Xylose 4TMS	28.06	1.78	1685	/
	Rhamnose 4TMS	28.83	1.77	1712	/
	Fructose 5TMS (anti)	33.01	1.76	1871	1867
	Fructose 5TMS (syn)	33.33	1.79	1882	1885
	Glucose 5TMS	33.46	1.82	1890	1898
	Mannose 6-phosphate 4TMS	43.09	2.16	2309	/
	Melibiose 8TMS	47.03	1.90	2505	2512
	Cellobiose 8TMS	50.40	1.90	2685	/
	Sucrose 8TMS	51.17	3.06	2726	2730
	Maltose 8TMS	51.47	1.93	2736	2732
	Galactinol 9TMS	55.25	2.06	2937	2943
Methylxanthines	Caffeine TMS	33.03	3.90	1872	1880
	Theobromine TMS	33.81	4.05	1902	/
Flavan-3-ols	Catechin 5TMS	53.33	2.41	2835	2840
	Epicatechin 5TMS	53.75	2.27	2863	/
	Gallocatechin 6TMS	54.33	2.17	2884	/
	Epigallocatechin 6TMS	54.50	2.32	2897	2903
Phenolic acids	Quinic acid TMS	32.50	1.85	1852	1853
	Gallic acid 3TMS	35.09	2.01	1954	1960
	Caffeic acid 3TMS	39.40	2.17	2136	2140
	Chlorogenic acid 6TMS	57.83	2.56	3074	3082
Others	(E)-Erythrono-1,4-lactone 2TMS	18.76	2.55	1375	1380
	Xylonic acid lactone TMS	26.42	2.50	1627	1627
	Ribono-1,4-lactone 3TMS	27.93	2.57	1680	1677
	Mannofuranose, 6-deoxy 4TMS	30.51	2.55	1776	/
	N-Acetyl-D-glucosamine 4TMS	37.92	2.32	2072	/
	Galactose oxime 6TMS	38.81	1.80	2110	/
	4-O-Coumaroyl-D-quinic acid, 5TMS	56.58	2.89	3008	3012

**Table 2:** Tea samples together with acronyms adopted in the text.

Origin	Harvest Year	Season	Elevation	Processing
Yunnan—YUN	2014	Spring—S	High elevation—H 1790 m	Pu′erh tea
	2015	Monsoon—M	Low elevation—L 1180 m	
Fujian—FUJ	2014	Spring—S	High elevation—H 690 m	Oolong tea
	2015	Monsoon—M	Low elevation—L 112 m	
Bigelow BIG	2015	May	-	Black tea
		July		
		August		
		October		

## Supplementary Materials

The following are available online: Figure S1: 2D data elaboration work-flow adopted to comprehensively map tea primary metabolites signatures, by adopting a combined untargeted and targeted UT template matching approach. Figure S2: schematic diagram of the extraction and derivatization procedure. Figure S3: experimental results on targeted primary metabolites extraction yields. Figure S4: method repeatability is indicated in terms of % relative standard deviation (%RSD) for both chromatographic dimensions (*^1^t_R_* and *^2^t_R_*) and for % response, collected from UT peak-regions.
