# Supplementary material for: Climate and Processing Effects on Tea (Camellia sinensis L. Kuntze) Metabolome: Accurate Profiling and Fingerprinting by Comprehensive Two-Dimensional Gas Chromatography/Time-of-Flight Mass Spectrometry
Source: Molecules. 2020 May 24;25(10):2447. doi: 10.3390/molecules25102447 (PMC7288030; doi:10.3390/molecules25102447)
Supplement: Supplementary file 1 [file molecules-25-02447-s001.pdf]

molecules

Supplementary material

Climate and Processing Effects on Tea (*Camellia sinensis* L. Kuntze) Metabolome: Accurate Profiling and Fingerprinting by Comprehensive Two-Dimensional Gas Chromatography/Time-of-Flight Mass Spectrometry

Federico Stilo [1], Giulia Tredici [1], Carlo Bicchi [1], Albert Robbat Jr. [2,*], Joshua Morimoto [2] and Chiara Cordero [1,*]

[1] Dipartimento di Scienza a Tecnologia del Farmaco, Università degli Studi di Torino, 10125 Turin, Italy; federico.stilo@unito.it (F.S.), giulia.tredici@edu.unito.it (G.T.); carlo.bicchi@unito.it (C.B.); chiara.cordero@unito.it

[2] Department of Chemistry, Tufts University, Medford, MA 02155, USA; albert.robbat@tufts.edu (A.R.J.); joshua.morimoto@tufts.edu

* Correspondence: albert.robbat@tufts.edu (A.R.); chiara.cordero@unito.it (C.C.); Tel.: +1-617-627-3473 (A.R.); Tel.: +39-011 6702197 (C.C.)

The supplementary material comprises four Supplementary Figures:

Figure S1: 2D data elaboration work-flow adopted to comprehensively map tea primary metabolites signatures, by adopting a combined Untargeted and Targeted UT template matching approach.

Figure S2: schematic diagram of the extraction and derivatization procedure.

Figure S3: experimental results on targeted primary metabolites extraction yields.

Figure S4: method repeatability is indicated in terms of % relative standard deviation (%RSD) for both chromatographic dimensions (1tR and 2tR) and for % response, collected from UT peak-regions.

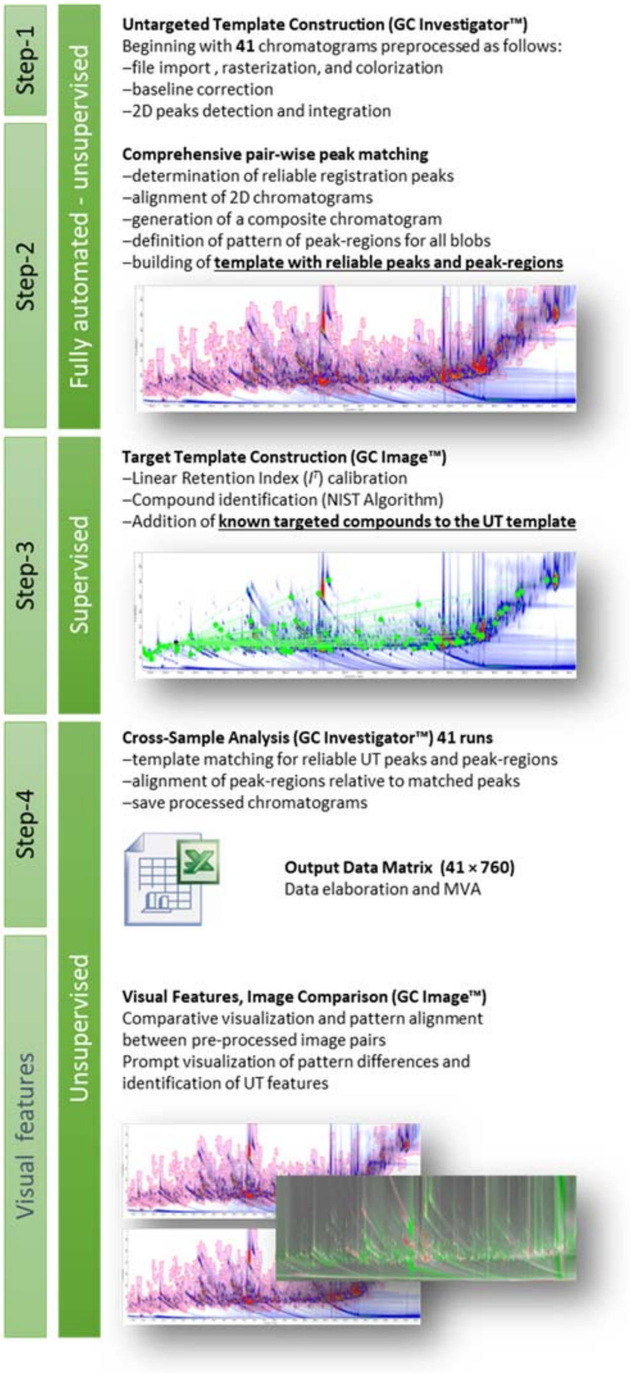

Figure S1. 2D data elaboration work-flow adopted to comprehensively map tea primary metabolites signatures, by adopting a combined Untargeted and Targeted UT template matching approach.

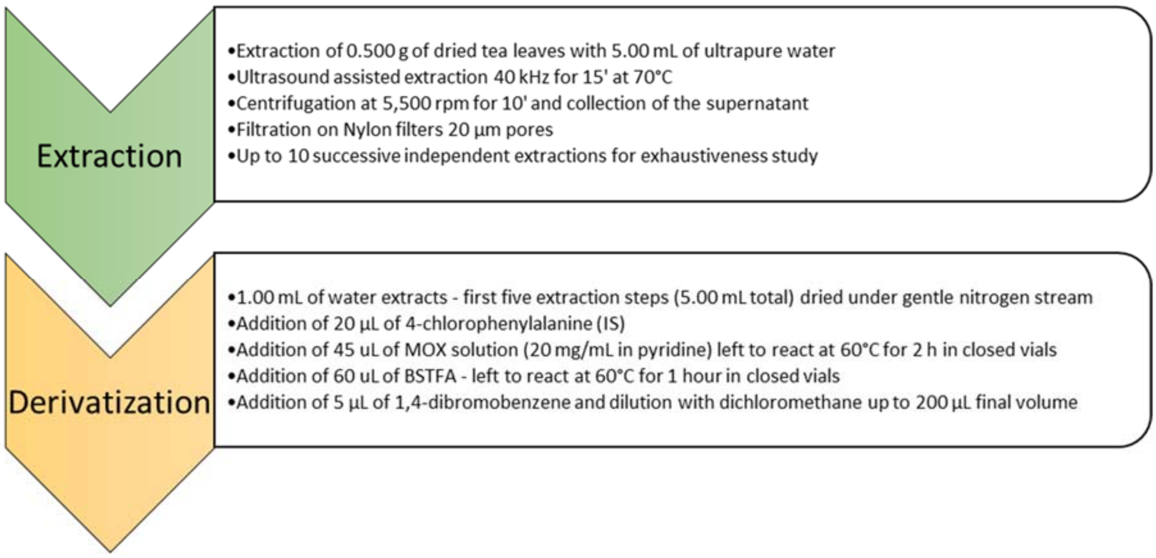

Figure S2. schematic diagram of the extraction and derivatization procedure.

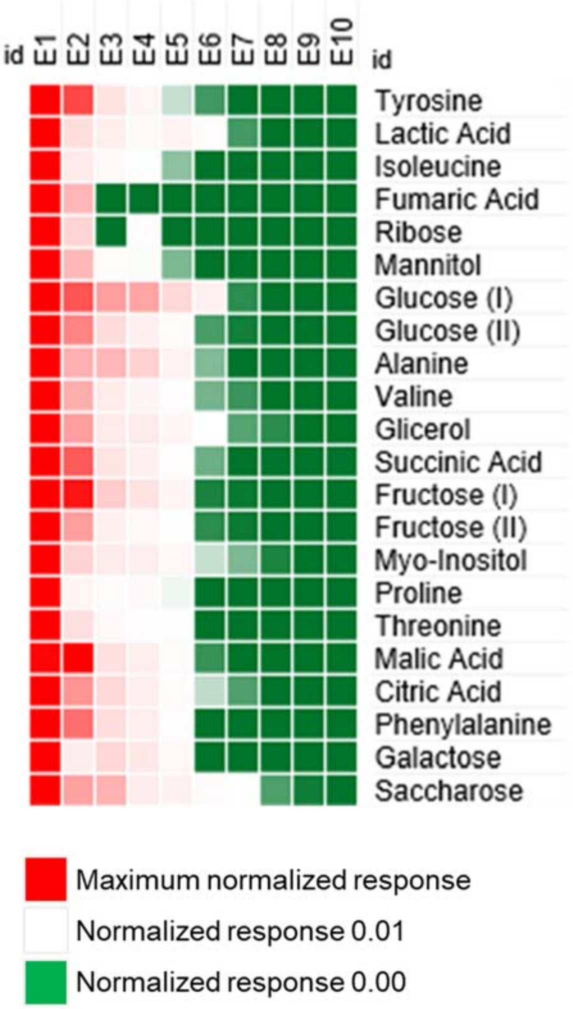

Figure S3. extraction yield results according to Figure S2 process; rows indicate relative metabolite concentration, while each column shows the relative amount by extraction step (from 1 to 10). All targeted compounds were extracted completely by step #9.

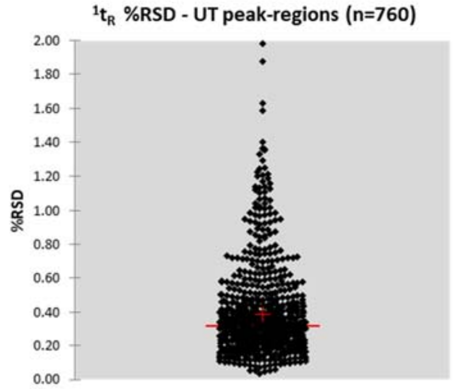

Statistic	%RSD
No. of observations	760
Minimum	0.0341
Maximum	1.9794
1st Quartile	0.2243
Median	0.3174
3rd Quartile	0.4492
Mean	0.3889
Variance (n-1)	0.0696
Standard deviation (n-1)	0.2638

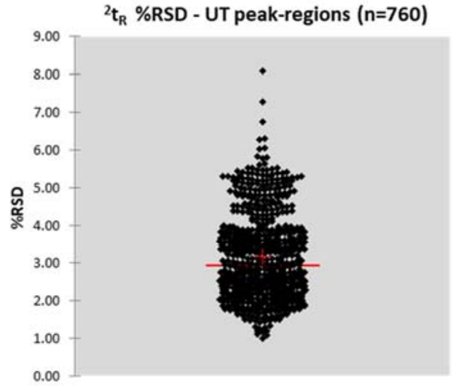

Statistic	%RSD
No. of observations	760
Minimum	0.9833
Maximum	8.0914
1st Quartile	2.2486
Median	2.9435
3rd Quartile	3.7709
Mean	3.1534
Variance (n-1)	1.2623
Standard deviation (n-1)	1.1235

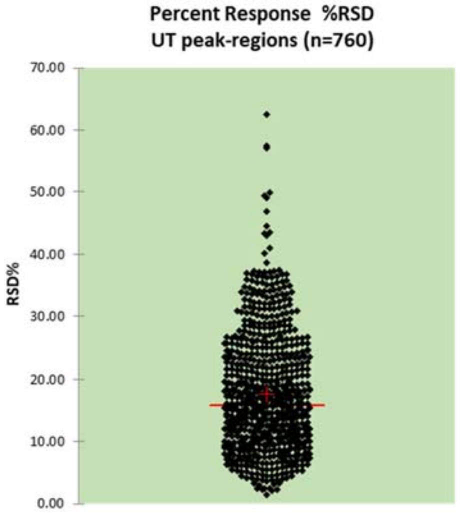

Statistic	RSD%
No. of observations	760
Minimum	1.4134
Maximum	62.4575
1st Quartile	10.6071
Median	15.8763
3rd Quartile	23.3992
Mean	17.6463
Variance (n-1)	88.3691
Standard deviation (n-1)	9.4005

Figure S4. method repeatability is indicated in terms of % relative standard deviation (%RSD) for both chromatographic dimensions (1tR and 2tR) and for % response, collected from UT peak-regions.

© 2020 by the authors. Licensee MDPI, Basel, Switzerland. This article is an open access article distributed under the terms and conditions of the Creative Commons Attribution (CC BY) license (http://creativecommons.org/licenses/by/4.0/).
